# Vertical transmission of dengue virus in the Yogyakarta airport area

**DOI:** 10.1186/s12199-018-0711-6

**Published:** 2018-06-05

**Authors:** Tri Baskoro Tunggul Satoto, Antok Listyantanto, Suzana Dewi Agustjahjani, Hari Kusnanto Josef, Barandi S. Widartono

**Affiliations:** 1 Center For Tropical Medicine, Faculty of Medicine, Universitas Gadjah Mada, Jl. Farmako Sekip Utara, Yogyakarta, Indonesia; 2 Port Health Quarantine, Adisucipto Airport, Yogyakarta, Indonesia; 3 Department of Public Health, Faculty of Medicine, Universitas Gadjah Mada, Yogyakarta, Indonesia; 4 Department of Geographic Science, Faculty of Geography, Universitas Gadjah Mada, Yogyakarta, Indonesia

**Keywords:** Transovarial Transmission Index, Airport, Dengue, *Aedes* spp.

## Abstract

**Background:**

International Health Regulations controls international travel including human movement, disease vector, and imported items to prevent the spread of dengue, especially in seaports, airports, and border crossing posts. This study aimed to determine dengue Transovarial Transmission Index (TTI) and distribution of dengue virus in the areas around Adisucipto Airport of Yogyakarta, Indonesia.

**Methods:**

The study was a descriptive analytic study with cross sectional design, conducted by mapping the spread of the dengue virus and identifying TTI in Adisucipto Airport. A total of 145 ovitraps were installed in both perimeter and buffer areas of the airport. Positive Ovitrap Index (OI), TTI, and serotype of dengue virus were examined. The TTI was identified using immunocytochemistry immunoperoxidase streptavidin biotin complex (IISBC) method in mosquito head squash preparations.

**Results:**

OI in the buffer area was 32 (45.1%), whereas OI in the perimeter area was 24 (32.4%). The TTI in the buffer and perimeter areas were 21 (18.3%) and 11 (18.9%), respectively. The TTI was found greater in the Aedes aegypti population compared to the Aedes albopictus population, both in the perimeter area (20% versus 16.7%) and the buffer area (20.3% versus 16.1%). Dengue virus serotype-2 (DENV-2) and dengue virus serotype-3 (DENV-3) were predominantly found in *Ae*. *aegypti* and *Ae*. *albopictus.*

**Conclusions:**

Buffer areas of Adisucipto Airport of Yogyakarta have higher risk as breeding sites for *Aedes* spp., predominantly DENV-2 and DENV-3 serotypes. High OI shows that the areas are likely to have higher risk of developing dengue outbreak.

## Background

In the last 50 years, the dengue disease has been spreading rapidly and extensively in the world following the geographical distribution to many countries, with the tendency of spreading from urban to rural areas. An estimated 50 million cases of dengue are found every year, and about 2.5 billion people are living in dengue endemic countries [1]. The World Health Organization (WHO) in 2005 issued a revision of the rules of international travel, known as the International Health Regulations (IHR) that included dengue as one of the diseases of Public Health Emergency of International Concern (PHEIC) that may have an impact on security problems of health and has the potential for causing an epidemic that can spread across borders between countries [1].

Tourists have a major role in the spread of dengue when entering areas inhabited with *Aedes* spp. mosquitoes [2], posing a risk of traveler viremia that could carry various strains of dengue serotypes. Study results showed an increasing trend of dengue cases per year (seasonal) with 522 travelers reported by The GeoSentinel Surveillance Network during the peak of dengue cases in Southeast Asia (June to September), Central Asia (October), South America (March), and The Caribbean (August, October), all of which indicate the relationship between travel and the occurrence of dengue epidemics [3]. In Southeast Asia, the annual morbidity rate has risen from 50 cases per 1000 travelers with dengue after traveling to a non-epidemic area to 159 cases per 1000 travelers during epidemics [3], although dengue transmission in the airport has not yet been reported until now.

The New Tokyo Narita International Airport Quarantine Post in Chiba Prefecture in year 2000 to 2002 had examined 233 passengers suspected of being infected with dengue virus: 1 case (4%) out of 26 cases identified in year 2000, 8 cases (12%) out of 69 cases identified in year 2001, and 22 cases (16%) out of 138 cases identified in year 2002 were confirmed as dengue infection [4]. Most of the passengers were infected after traveling from Southeast Asian and South Asian countries, one from African countries, one from Central American countries, one from Central and South American countries, and one from South American countries [4]. Fever screening in Taiwan Airport begun in July 2003 to June 2004 identified 40 dengue cases, among which 33 people (82.5%) were confirmed as viremic patients [5]. During 2007 to 2010, sentinel surveillance in Taiwan Airport showed that most of the dengue-infected travelers had just returned from endemic areas around the Southeast Asian region, namely Indonesia (21.0 to 35.1%), Vietnam (20.1 to 32.0%), Thailand (5.0 to 13.0%), the Philippines (9.0 to 12.3%), Cambodia (4.1 to 8.0%), Malaysia (2.0 to 4.1%), Singapore (1.1 to 3.4%), India (0 to 1.1%), and only few travelers who had just returned from South America (0 to 0.7%) [6]. In September 2013, in Germany, a traveler who had just returned from Japan was confirmed as having type 2 dengue virus infection; hence, the German Health Authority performed strict monitoring towards travel history of the travelers in order to evaluate risk potential of travelers having dengue virus infection [7].

International travel such as human movement, disease vector, and contaminated items that potentially cause widespread disease are regulated by the WHO in the International Health Regulations 2005 Article 9 (nine). Each country is required to perform dengue risk assessment to prevent the spread of dengue between countries by strengthening surveillance and supervision at the entrance areas, i.e., seaports, airports, and border crossing posts [8].

According to the Law Act 1 Year 1962 regarding Marine Quarantine and Act 2 Year 1962 regarding Sky Quarantine, as well as the IHR Year 2005 Article 2, all of which state that seaports and airports are obliged to be free from disease vectors and are required to perform disease control and prevention suitable to the potential risk factors without interfering with the commercial traffic [8]. Perimeter ports including seaports, airports, and rural ports are expected to be free from both larval stage and adult stage of *Aedes aegypti* mosquitoes, whereas in the buffer areas the following values are considered essential: House Index (HI) of less than, or equal to 1%, Breteau Index (BI) of < 50, biting rate of < 2.5, and OI of < 15% [9].

This study was conducted to acquire information regarding the presence of *Aedes* spp. mosquitoes and the extent of transovarial transmission in the perimeter and buffer areas of Yogyakarta’s Adisucipto Airport. The study was also intended to determine dengue Transovarial Transmission Index (TTI) and distribution of dengue virus in areas in and around Adisucipto Airport, Yogyakarta, Indonesia.

## Methods

This research is a descriptive analytic study with a cross sectional design. The study was conducted by mapping the spread of dengue virus and identifying TTI in Adisucipto Airport of Yogyakarta, Indonesia (07^°^ 47′ 17″ S and 110^°^ 25′ 54″ E). The study was done in December 2015 to May 2016, during which rainy season took place.

Dengue cases were collected based on data retrieved from local primary health center. All of dengue cases were diagnosed with dengue non-structural protein 1 (NS-1) antigen test. However, serotypes of infecting dengue virus were unknown. The study was conducted by installing 145 ovitraps in both the perimeter and buffer areas of the airport. We defined the zones as the following definitions: perimeter area is 100-m peripheral area from airport apron; buffer area is 400-m peripheral area from airport apron [9]. The study could only be performed at the northern and western area of the airport due to limited public access to the eastern and southern area.

Ovitrap Index (OI) is calculated from number of ovitraps with positive eggs divided by sum of ovitraps installed. Ovitraps were installed outdoors and indoors within 35–50 m distance. Positive OI and TTI of dengue virus were examined later on. After colonization of *Aedes* spp. eggs, TTI was identified using immunocytochemistry immunoperoxidase streptavidin biotin complex (IISBC) method in mosquito head squash preparations [10]. Rate of TTI was obtained by dividing the number of DENV-positive IISBC samples by total number of IISBC samples examined and was expressed as percentage [10]. The virus was identified through serotype examination by Lanciotti’s RT-PCR. Transovarial transmission in both *Aedes aegypti* and *Aedes albopictus* mosquitoes obtained from indoor and outdoor ovitraps were compared. The analysis of OI distribution was conducted using the software ArcGIS 9.2 (Esri, New York) to provide mapping and spatial reasoning to yield location-based data. The data were entered into nearest neighbor analysis, resulting in the distance between each feature centroid and its nearest neighbor’s centroid location. The *z*-score and *p* value are measures of statistical significance that are used in determining whether or not to reject the null hypothesis: features are randomly distributed.

The presence of high TTI value is considered an important mechanism for the maintenance of the virus in nature and may be associated in the occurrence of dengue epidemics and outbreak. High OI supports the potential of dengue outbreaks as well [11].

## Results

**Fig. 1:**
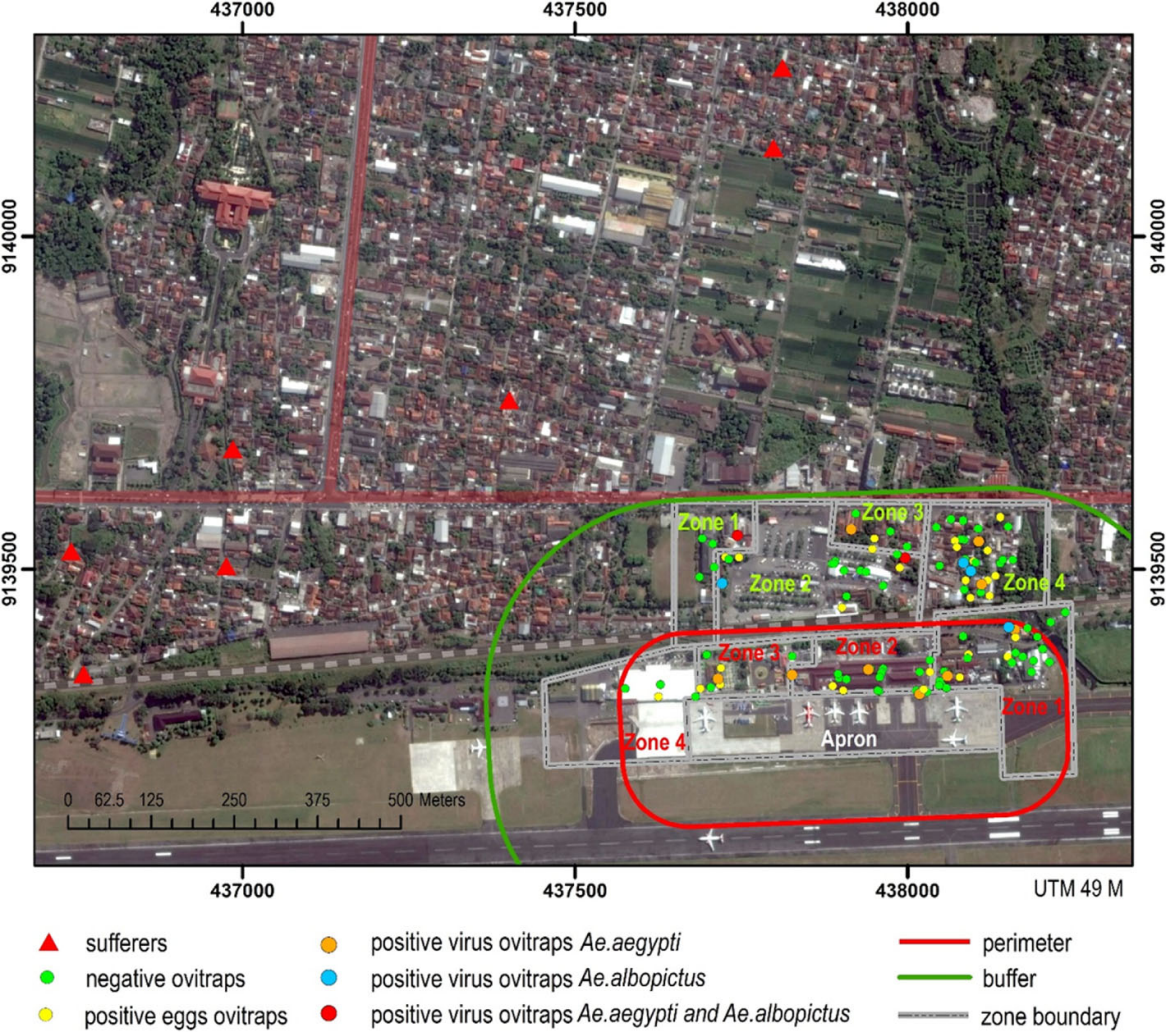
Distribution map of dengue virus and dengue cases in Adisucipto Airport Yogyakarta in every Zones. Four zones in perimeter area are cargo log (zone 1), terminal A (zone 2), office (zone 3), and terminal B (zone 4), while four areas in buffer area (**—**) are terminal B (zone 1), parking and public facilities (zone 2), neighborhood 7 (zone 3), and neighborhood 8 (zone 4). Buffer area has more potential as breeding sites of *Aedes* spp. mosquitoes. The distribution of *Aedes* spp. mosquitoes based on positive Ovitrap Index in Yogyakarta’s Adisucipto Airport was found evenly distributed in the area, positive eggs ovitraps, positive virus ovitraps *Ae*. *aegypti*, positive virus ovitraps *Ae*. *albopictus*, and positive virus ovitraps *Ae*. *aegypti* and *Ae*. *albopictus*, with the highest number of positive OI found in the zone 4, the neighborhood 8 (RT 8) of village of Maguwoharjo. Negative ovitraps and dengue patients (sufferers) distribution may also be observed

Positive OI of *Aedes* spp. mosquitoes in the buffer area was 32 (45.1%), with outdoor ovitraps showing greater proportion (50%) compared to indoor ovitraps (37.9%). OI in the buffer area was 32 (45.1%), whereas OI in the perimeter area was 24 (32.4%) as shown in Table 1. The distribution of *Aedes* spp. mosquitoes based on positive OI in Yogyakarta’s Adisucipto Airport was found evenly distributed in the surrounding areas, with the highest number of positive OI found in the zone 4, the neighborhood 8 (*Rukun Tetangga/RT 8*) of village of Maguwoharjo (14 ovitraps or 9.6%), and the least number of positive OI found in zone 4 of perimeter area, the B Terminal (2 ovitraps or 1.4%) as shown in Fig. 1.

**Table 1:** Distribution of ovitrap installation in Adisucipto Airport of Yogyakarta

Location	Situation		(+) Eggs		OI (%)	
	Indoor	Outdoor	Indoor	Outdoor	Indoor	Outdoor
Perimeter area	40	34	12	12	30	35.3
Total number	74		24		32.4	
Buffer area	29	42	11	21	37.9	50
Total number	71		32		45.1

The nearest neighbor analysis shows that if the *z-*score is less than 1, the pattern exhibits clustering, whereas if the index is greater than 1, the trend is towards dispersion. The analysis of OI distribution resulted in *z*-score of − 6.1 and *p* value of < 0.001 that indicates the tendency of clustered OI distribution. The shortest distance of positive ovitraps was 3.6 m, while the farthest distance was 19 m with average distance of 13.2 m. The results indicate that the distance is still within the range of mosquito disease transmission.

Through identification by examination using immunohistochemistry (IHC) method, as many as 11 samples (18.9%) in the perimeter area and 21 samples (18.3%) in the buffer area of Yogyakarta’s Adisucipto Airport were found positive for transovarial transmission. The transovarial transmission was observed higher in percentage in *Aedes aegypti* mosquitoes, with 20 to 16.7% in the perimeter area and 20.3 to 16.1% in the buffer area (Table 2). In *Aedes aegypti* mosquitoes, positive transovarial transmissions were more commonly detected in samples obtained from indoor-installed ovitraps: 23.5 to 17.4% in the perimeter area and 25 to 14.8% in the buffer area. On the other hand, positive transovarial transmissions in *Aedes albopictus* mosquitoes were all found in samples from outdoor-installed ovitraps, with 16.7% in the perimeter area and 16.1% in the buffer area. Unfortunately, we did not perform further analysis to identify statistical significance of this difference in findings between mosquito species. Distribution of dengue virus in the Adisucipto Airport of Yogyakarta was predominantly found in zone 4 of the buffer area, the RT 8 of village of Maguwoharjo (4 cases or 12.5%), and was least found in zone 2, the parking lot and common facility, and zone 3, RT 7 of village of Maguwoharjo, with both areas having only 1 case detected (3.1%). Dengue virus distribution tends to follow the distribution pattern of ovitraps. In both perimeter and buffer areas of the Adisucipto Airport, there were no dengue cases found. As described in Fig. 1, the shortest distance of dengue patients was 389 m, whereas the farthest distance was 1050 m.

**Table 2:** Analysis of transovarial transmission of *Aedes* spp. mosquitoes in Adisucipto Airport of Yogyakarta

Location	Immunohistochemistry (IHC)		Transovarial transmission		% transovarial transmission	
	*Ae*. *aegypti*	*Ae*. *albopictus*	*Ae*. *aegypti*	*Ae*. *albopictus*	*Ae*. *aegypti*	*Ae*. *albopictus*
Perimeter area	40	18	8	3	20	16.7
Total number	58		11		18.9	
Buffer area	59	56	12	9	20.3	16.1
Total number	115		21		18.3	

The analysis of dengue virus distribution was conducted using the nearest neighbor analysis and resulted in *z*-score of − 2.0 and *p* value of 0.04 that indicates the tendency of clustered distribution. The shortest distance of positive ovitraps was 3.1 m, while the farthest distance was 33 m with an average distance of 15.2 m.

**Fig. 2:**
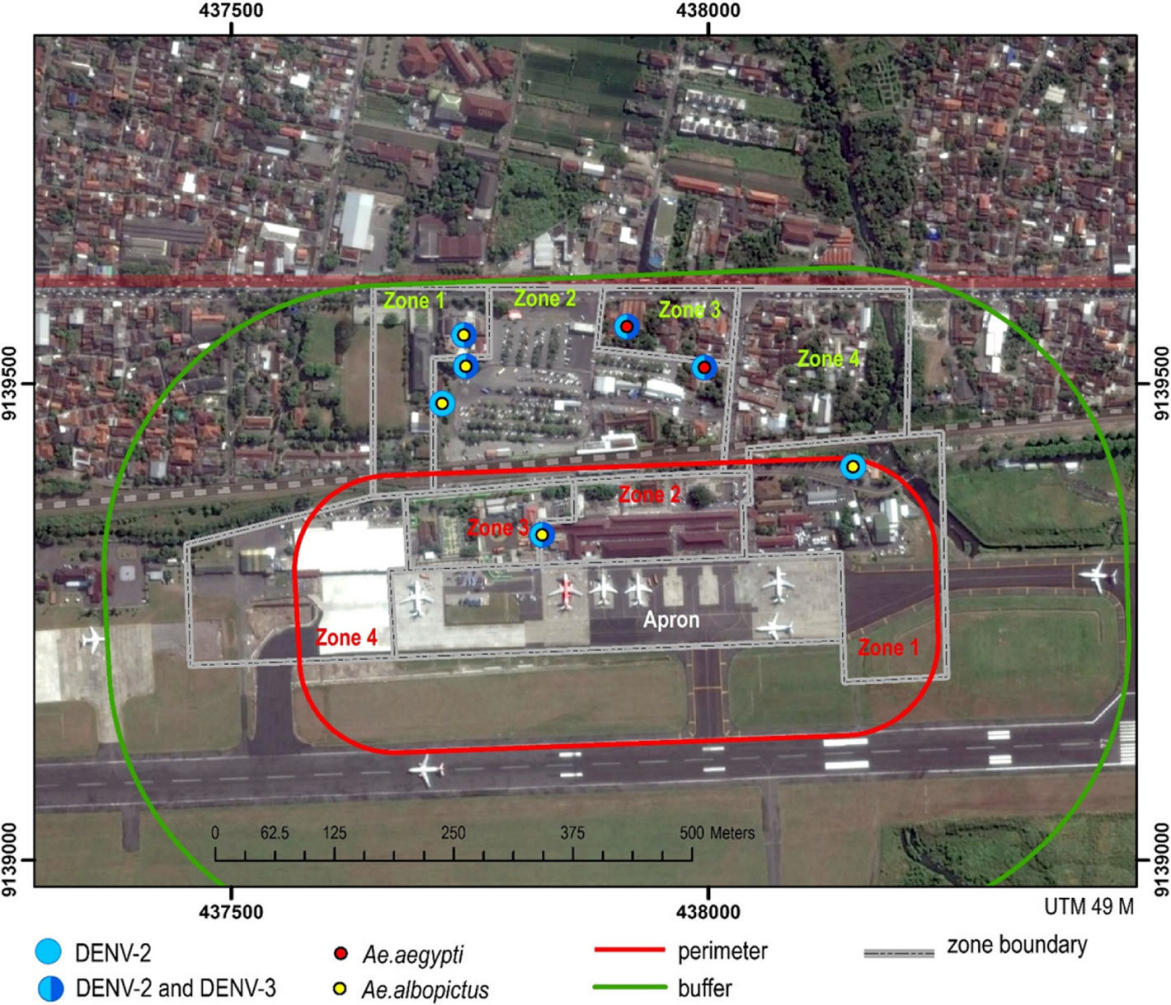
Distribution map of dengue virus and dengue cases in Adisucipto Airport Yogyakarta. Four zones in perimeter area are cargo log (zone 1), terminal A (zone 2), office (Zone 3), and terminal B (zone 4), while four areas in buffer area are terminal B (zone 1), parking and public facilities (zone 2), neighborhood 7 (zone 3), and neighborhood 8 (zone 4). One spot was positive in perimeter area for DENV-2 and one spot positive for DENV-2 and DENV-3, both of which were also positive for *Ae*. *albopictus*. One spot was positive in buffer area for DENV-2 and two spots were positive for DENV-2 and DENV-3, all of which were also positive for *Ae*. *albopictus*. There were two spots positive in buffer area for DENV-2, DENV-3, and *Ae*. *aegypti*

Dengue virus serotype examination was performed in dengue-positive *Aedes* spp. mosquito-colonized samples and in positive transovarial transmission samples, all of which were coded in species and location. The serotype examination was conducted using Lanciotti’s RT-PCR method and resulted in the detection of DENV-2 and DENV-3 serotypes in *Aedes aegypti* and *Aedes albopictus* mosquitoes in Adisucipto Airport of Yogyakarta (Table 3). Data analysis on dengue virus serotype distribution in Yogyakarta’s Adisucipto Airport resulted in *z*-score of 1.4 and *p* value of 0.14 that indicates tendency of random dengue virus serotype distribution. The shortest distance of dengue virus serotype was 33 m, while the farthest distance was 173 m with average distance of 94.1 m as shown in Fig. 2.

**Table 3:** Serotypes of dengue virus in *Aedes* spp. mosquitoes in Adisucipto Airport of Yogyakarta

Location	RT-PCR		DENV serotype
	*Aedes aegypti*	*Aedes albopictus*	
Perimeter area			
17a		12	DENV 2
54a		2	DENV 2 and 3
Buffer area			
110a	12		DENV 2 and 3
119a	5		DENV 2 and 3
132a		7	DENV 2 and 3
134a		4	DENV 2 and 3
136a		2	DENV 2

## Discussion

Ovitrap Index (OI) in the buffer area was higher compared to that in the perimeter area. The results showed that the buffer areas have more potential as breeding sites of *Aedes* spp. mosquitoes. The distribution of *Aedes* spp. mosquitoes based on positive OI in Yogyakarta’s Adisucipto Airport was found evenly distributed in the surrounding areas, with the highest number of positive OI found in the zone 4, the neighborhood 8 (*Rukun Tetangga/RT 8*) of village of Maguwoharjo. OI of < 5% shows that the distribution of *Aedes* spp. mosquitoes is least spread, while OI of 5–20% shows that the *Aedes* spp. mosquitoes are moderately spread in the study area [12]. OI of < 10% indicates that the area has no risk in the development of dengue outbreak, whereas OI of > 10% indicates that the area has the potential for developing dengue outbreak [13]. In the area of Yogyakarta’s Adisucipto Airport, OI of 45.1% was found, which indicates potential for developing dengue outbreak.

The analysis of OI distribution resulted in *z*-score of − 6.1 and *p* value of < 0.001 that indicates the tendency of clustered OI distribution. Through identification by using IHC method, the transovarial transmission was observed higher in percentage in *Aedes aegypti* mosquitoes in the perimeter area compared to that in the buffer area. The percentage alone presumes that *Aedes aegypti* mosquitoes may have a more dominant role in the transmission of dengue in the area of Adisucipto Airport of Yogyakarta. A similar study in Manado, Indonesia, also showed that the TTI in *Aedes aegypti* mosquitoes is significantly higher than that in *Aedes albopictus* mosquitoes [14]. Previous research established that *Aedes aegypti* mosquitoes have a major role in human dengue transmission [15]. A TTI rate of 20% allows the maintenance of highly stable vertical infection that persists for several generations [16].

Positive transovarial transmissions in *Aedes aegypti* mosquitoes were more commonly detected in samples obtained from indoor-installed ovitraps in the perimeter area. The results suggest that *Aedes aegypti* mosquitoes tend to do oviposition indoor rather than outdoor. A study in three localities in Malaysia also showed that ovitraps were more likely to yield positive results if installed indoor [17].

Positive transovarial transmissions in *Aedes albopictus* mosquitoes were all found in samples from outdoor-installed ovitraps. Distribution of dengue virus in the Adisucipto Airport of Yogyakarta was predominantly found in zone 4 of the buffer area, the RT 8 of village of Maguwoharjo. Dengue virus distribution tends to follow the distribution pattern of ovitraps. In both perimeter and buffer areas of the Adisucipto Airport, there were no dengue cases found. This result may be due to lack of good host immunity, higher activities of humans in the airport so that the mosquitoes were not able to bite very often [18], and low number of *Aedes* spp. mosquitoes in the Adisucptio Airport: 378 mosquitoes in ± 560,000 m^2^ perimeter and buffer areas. Other possible contributing factor is low TTI of dengue virus (18.9% in perimeter area and 18.3% in buffer area). These TTI values are considered low as the study was conducted in endemic area and in rainy season, during which dengue transmission is on its peak; therefore TTI values should exceed 30% [19]. As described in Fig. 1, the shortest distance of dengue patients was 389 m, whereas the farthest distance was 1050 m.

The analysis of dengue virus distribution was conducted using the nearest neighbor analysis and resulted in a *z*-score of − 2.0 and *p* value of 0.04 that indicates the tendency of clustered distribution. The shortest distance of positive ovitraps was 3.1 m, while the farthest distance was 33 m with average distance of 15.2 m. Although no specific threshold values have been established for each arbovirus, absence of severe dengue cases in Thailand was noted when the density of *Ae*. *aegypti* eggs per ovitrap per week was less than two [20]. Moreover, despite using a different ovitrap, DENV transmission occurred in Taiwan when the density of eggs per house (two ovitraps per house) was around two [21]. In determining transmission of dengue virus, TTI is important in showing that dengue virus from infected female mosquitoes may be spread into the ovarii and transmitted to the next generation, whereas OI correlates with mosquito population and represents true mosquito infestations in the area [11, 22].

Dengue virus serotype examination was performed in dengue-positive *Aedes* spp. mosquito-colonized samples and in positive transovarial transmission samples, all of which were coded in species and location: DENV-2 and DENV-3. Similar results were obtained in Bantul, Indonesia, which showed the predominant serotypes of dengue virus in *Aedes aegypti* mosquitoes were DENV-3 (12 locations), followed by DENV-2 (3 locations), DEN-4 (1 location), and DENV-1 (0 location) [23]. Studies conducted in Thailand [24] and Singapore [25] showed that DENV-2 was the predominant serotype found. In areas with high dengue endemicity, the predominant serotypes were DENV-2 and DENV-3, while in areas with low dengue endemicity the prevalent serotypes were DENV-1 and DENV-2 [21]. A similar study conducted in 2007 to 2009 in Brazil reported that the highest number of mosquito larvae found was *Aedes aegypti* (3417 mosquitoes or 91%), followed by *Aedes albopictus* (336 mosquitoes or 9%), with detected serotypes of DENV-3 *Aedes albopictus*, DENV-2 *Aedes aegypti*, and DENV-2 and DENV-3 *Aedes albopictus* [26].

The study has several limitations. First, the study was conducted only once during rainy season, which would have been better if performed simultaneously in other season as well. Second, study area was limited only to areas open for public access, not including those areas that belonged to the Air Force and not open for public.

## Conclusion

The study shows that buffer areas of Adisucipto Airport of Yogyakarta have higher risk as breeding sites for *Aedes* spp. mosquitoes, predominantly DENV-2 and DENV-3 serotypes. OI of 45.1 indicates that the area has the potential for developing dengue outbreak.

## Abbreviations

BI: Breteau Index
DENV-1: Dengue virus serotype-1
DENV-2: Dengue virus serotype-2
DENV-3: Dengue virus serotype-3
DENV-4: Dengue virus serotype-4
HI: House Index
IHR: International Health Regulations
IISBC: Immunocytochemistry immunoperoxidase streptavidin biotin complex
NS-1: Non-structural protein 1
OI: Ovitrap Index
PHEIC: Public Health Emergency of International Concern
RT: *Rukun Tetangga* (neighborhood)
RT-PCR: Reverse transcriptase-polymerase chain reaction
TTI: Transovarial Transmission Index
WHO: World Health Organization
